# Predicting the crystal structure of $\hbox {N}_5\hbox {AsF}_6$ high energy density material using ab initio evolutionary algorithms

**DOI:** 10.1038/s41598-021-86855-2

**Published:** 2021-04-12

**Authors:** El Mostafa Benchafia, Xianqin Wang, Zafar Iqbal, Sufian Abedrabbo

**Affiliations:** 1 Department of Physics, Khalifa University, Abu Dhabi, UAE; 2 Department of Chemical, Biological and Pharmaceutical Engineering, New Jersey Institute of Technology, Newark, NJ 07102 USA; 3 Department of Chemistry and Environmental Science, New Jersey Institute of Technology, Newark, NJ 07102 USA; 4 Department of Physics, University of Jordan, Amman, Jordan

**Keywords:** Chemistry, Physics

## Abstract

$\hbox {N}_5\hbox {AsF}_6$ is the first successfully synthesized salt that has a polymeric nitrogen moeity ($\hbox {N}_5^+$). Although 12 other $\hbox {N}_5^+$ salts followed, with $\hbox {N}_5\hbox {SbF}_6$ and $\hbox {N}_5\hbox {Sb}_2\hbox {F}_{11}$ being the most stable, the crystal structure of $\hbox {N}_5\hbox {AsF}_6$ remains unknown. Currently, it is impossible to experimentally determine the structures of $\hbox {N}_5\hbox {AsF}_6$ due to its marginal stability and explosive nature. Here, following an ab initio evolutionary prediction and using only the stoichiometry of $\hbox {N}_5\hbox {AsF}_6$ as a starting point, we were able to reveal the crystal structure of this high energy density material (HEDM). The $\hbox {C}_{2V}$ symmetry of the $\hbox {N}_5^+$ cation, as suggested from earlier investigations, is confirmed to be the symmetry adopted by this polymeric nitrogen within the crystal. This result gave full confidence in the validity of this crystal prediction approach. While stability of the $\hbox {N}_5^+$ within the crystal is found to be driven by electronic considerations, the marginal stability of this HEDM is found to be related to a partial softening of its phonon modes.

## Introduction

Polymeric nitrogen (PN), a form of singly or doubly bonded nitrogen atoms as clusters or extended solid networks, has been the subject of plethora of theoretical and experimental investigations. The interest in these materials originate from their energy storage capabilities. With bonding energies at: 160 kJ mole$^{-1}$ for nitrogen single bond N–N, 418 kJ mole$^{-1}$ for nitrogen double bond N$=$N and 954 kJ mole$^{-1}$ for nitrogen triple bond N$\equiv$N^1^, an energy release of 794 kJ mole$^{-1}$ could be harnessed from the single to the molecular triple bond transformation. This is equivalent to tenfold higher detonation pressures compared to the high energy explosive HMX^2,3^. The pioneering work of McMahan and Lesar^4^ in 1985 stands among the first attempts aiming at investigating the existence of polymeric nitrogen structures. They predicted that the transition pressures from molecular to monoatomic nitrogen was less than 1 Mbar. This was well within reach of most high pressure anvil cells in use and the simple cubic structure formed showed the common distortions found in group V elements. In 1992, McMahan et al.^5^ theoretically identified more extended nitrogen solids at relatively low pressures that are within reach of typical diamond anvil cells. Notable among these structures is the cubic gauche phase with threefold coordinated nitrogen atoms adopting all-gauche dihedral angles. A 2004 experimental breakthrough by Eremets et al.^6^ succeeded in synthesizing this structure by pressurizing molecular nitrogen above 110 GPa at temperatures above 2000 K by laser heating in a diamond anvil cell. Another polymeric nitrogen structure in the form of two $\hbox {N}_8$ isomers per unit cell held together by van der Walls forces was predicted by Hirshberg et al.^7^ but has yet to be synthesized. The calculations showed that this polynitrogen form is stable even at ambient pressure and is more stable than the cubic gauche phase below 20 GPa. There is a strong debate whether the synthesis of polymeric nitrogen could be achieved beyond out-of-equilibrium processes, such as high pressure combined with high temperature and plasma methods^6,8^. To this date, extended solid forms of polymeric nitrogen were only obtained under extreme conditions and in most cases the structures were lost upon releasing pressure. To this date, extended solid forms of polymeric nitrogen were only obtained under extreme conditions and in most cases the structures were lost upon releasing pressure. Cluster polynitrogens on the other hand had substantial success with the pentazolium $\hbox {N}_5^+$ cation stabilized as the $\hbox {N}_5\hbox {AsF}_6$ salt in the work of Christe et al.^9^. This received a lot of attention in the scientific community as well as in many media outlets, such as the New York Time (see Supplementary Note 1) and also led to substantial sponsorship of polynitrogen chemistry^10^ by the Defense Advanced Research Projects Agency (DARPA). Marginal stability of the $\hbox {N}_5\hbox {AsF}_6$ solid warrants explosive hazard possibilities that prohibits the use of X-ray diffraction to determine its crystal structure. Thus, no known crystal structure of this compound exists. Attempts to clarify the thermal stability of $\hbox {N}_5^+$ salts can be found in the work of Yu^11^, where the relative stability of the $\hbox {N}_5^+$ salts is attributed to the role played by the central atom of the counter anion (e.g. As, Sb. Al, B $\ldots$ etc) and their ligands (e.g. F, (OH)$_4\hbox {F}_2$, ($\hbox {CF}_3$)$_4 \ldots$ etc). To the best of our knowledge, the only known crystal structure of a $\hbox {N}_5^+$ salt is that of $\hbox {N}_5\hbox {Sb}_2\hbox {F}_{11}$, which was synthesized using the $\hbox {N}_5\hbox {SbF}_{6}$ precursor^12^ (The crystal arrangement of $\hbox {N}_5\hbox {Sb}_2\hbox {F}_{11}$ can be found in Supplementary Fig. 1). Fully understanding polymeric nitrogen salts such as $\hbox {N}_5\hbox {AsF}_6$ will shed more light and guide the experimental search for the long-sought-after polymerization of nitrogen. For example, in light of the most recent synthesis of cyclic $\hbox {N}_5^-$ in ($\hbox {N}_5$)$_6$($\hbox {H}_3$O)$_3$($\hbox {NH}_4$)$_4$Cl^13^, a $\hbox {N}_{10}$ synthesis from $\hbox {N}_5^+$ and $\hbox {N}_5^-$ precursors is worth investigating. In this paper, using an evolutionary ab initio search procedure, we were able to obtain the structure of $\hbox {N}_5\hbox {AsF}_6$. We therefore performed a thorough investigation into the overall stability of the crystal from a phonon perspective. We also assessed $\hbox {N}_5^+$ stability within the crystal from an electronic perspective.

Despite the tremendous success of evolutionary search techniques in the last 10 years in predicting superconductors^14–17^, magnetic materials^18,19^and novel compounds that defy conventional chemistry^20,21^, the work of Pakhnova et al.^22^ stands out as the only predictive search fully dedicated to the study of energetic materials including most known explosives such as TNT, PETN, $\beta$-HMX, CL-20, TATB and their mixtures. Considering that clusters should retain chemical stability in the crystal as a requirement for the overall stability of the compound^7^, the crystal prediction methodologies will identify polynitrogens not only in the gas phase, but it will also help in identifying the best clusters and their embodiment with other elements across the periodic table. In this report, we adopted these well-established evolutionary search methodologies to the energetic material $\hbox {N}_5\hbox {AsF}_6$ salt. Quantum mechanics ab initio calculations that can span from hundreds to thousands of possible structures are then used to relax the structures and survival of the fittest leads the search towards the true structure.

## Results and discussion

### Structure search

**Figure 1:**
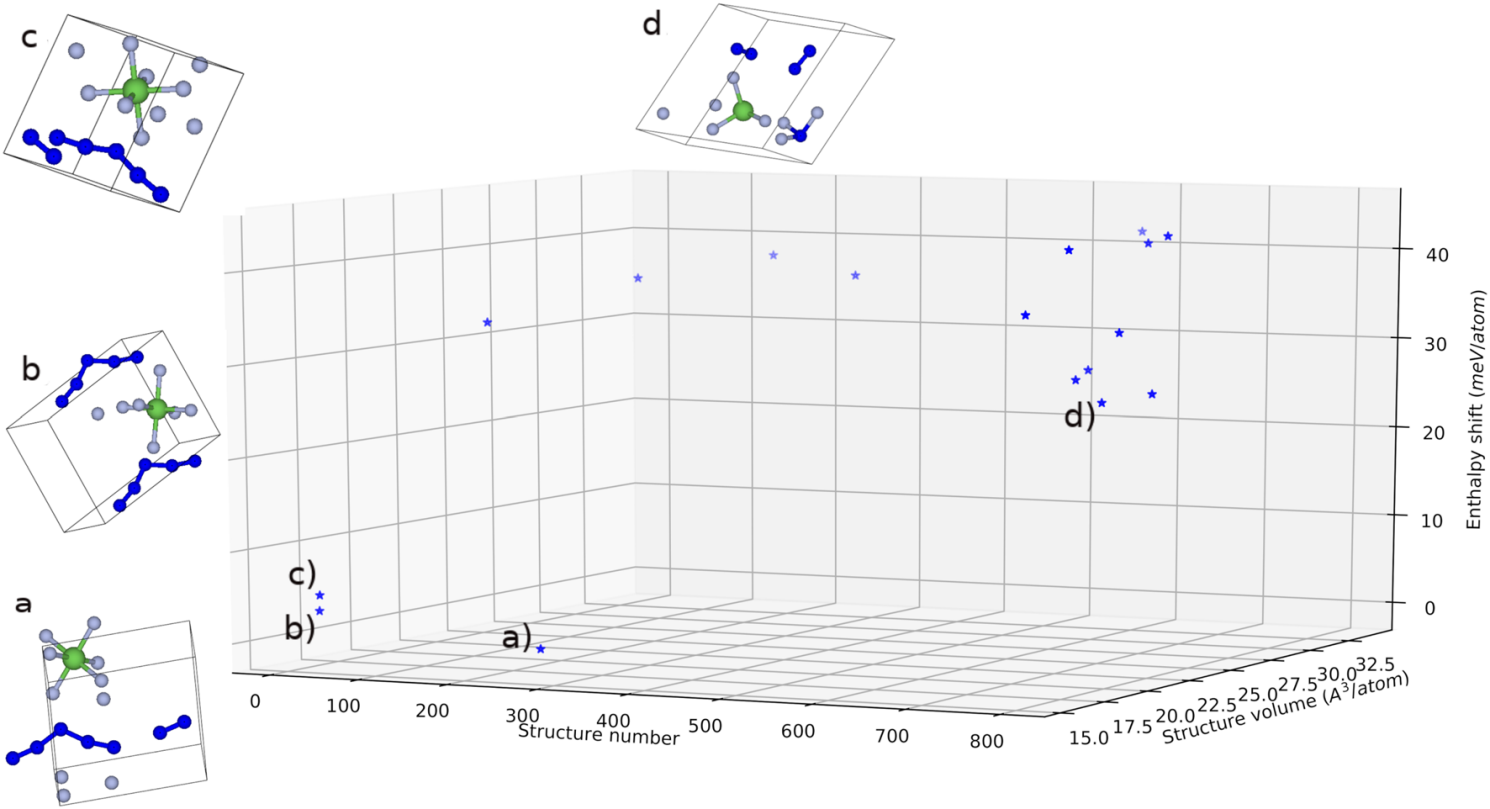
3-D plot depicting the overall evolutionary search performed with the USPEX^23–25^ code and quantum espresso DFT^26,27^ engine for geometry optimization. 28 Generations with a total of 790 crystal structures were produced for optimization. For clarity only the best 16 structures were plotted against their structure number along the search, their corresponding volume and enthalpy. Ball and stick representation of the best four structures (**a**–**d**) are presented along with their position in the enthalpy/volume V-H landscape. The enthalpy shift is from the best structure (structure (**a**)) in meV/atom).

The search for the crystal structure of $\hbox {N}_5\hbox {AsF}_6$ was carried out using the evolutionary algorithm for structure predictions as developed in the code USPEX^23–25^. A single unit with five nitrogens, one arsenic and six fluorine atoms and with no other chemical or structural knowledge set the basis for the calculations. In the course of a typical search, generations of structures were produced by the USPEX code and geometry optimization was conducted on each structure using density functional theory (DFT). As the first produced generations are more likely to be unphysical, geometry optimization often fails to converge. Additionally, nitrogen and fluorine in $\hbox {N}_5\hbox {AsF}_6$ structures represent yet another challenge due to their comparable ionic size and electronegativity. Hence, computational compromises are needed to avoid too many failing structures leading to the halt of the evolutionary search. Another hurdle is the inherent marginal stability of the compound itself as reported in its synthesis and characterization procedures. Low temperature Raman and IR vibrational frequencies as well as DFT calculations hypothesized the $\hbox {C}_{2V}$ symmetry of the $\hbox {N}_5^+$ cation^9,12^. In this work, the zero temperature, zero pressure evolutionary calculations succeeded in producing this $\hbox {C}_{2V}$ polymerization of nitrogen in the best three structures as shown in Fig. 1a–c; thus, representing a validation of the methodology used. In addition, the AsF6$^-$ anion octahedral $\hbox {O}_h$ symmetry was also obtained in the best three predicted structures.

The nitrogen $\hbox {C}_{2V}$ as well as the $\hbox {AsF}_6^{-}$ configurations are lost in the fourth best structure that is shown in Fig. 1d. In this structure, the system exhibits a decomposition into molecular $\hbox {N}_2$, nitrogen trifluoride $\hbox {NF}_3$ and arsenic trifluoride $\hbox {AsF}_3$. Moreover, the aforementioned fourth structure, where the $\hbox {N}_5^+$ polymeric nitrogen is lost, is also far in its enthalpy from the first three structures. The energy shift of the fourth best structure is as high as 26.3 meV/atom from the best structure. The shift, however, is only 3.1 meV/atom and 5.1 meV/atom from the second and third best structures, respectively. The three best structures also set themselves as a separate subset of structures in the volume landscape with a volume of $\sim$16 Å$^3$/atom. This is among the lowest volumes found in comparison to all the structures obtained along the whole evolutionary search procedure with the USPEX^23–25^ algorithm. The rest of the unfavorable structures possess higher volumes than this special set. This suggests the stability of the $\hbox {N}_5^+$ in this solid at relatively smaller volumes and confirms the well-known polymorphism of polymeric nitrogen structures like the singly bonded cubic gauche to form at higher pressures.

The best energetically favorable structure shown in Fig. 1a (different orientations of the structure are shown in Supplementary Fig. 2 ), is found to be triclinic of space group H1 P1 (Crystallographic Information File (CIF) provided in Supplementary Note 2) based on DFT calculations with PBEsol^28^ general gradient approximation (GGA)^29^. In fact, neither PBE GGA nor LDA^27^ approximations were capable of handling the evolutionary search. This is due to the computational difficulty involved in configuring an odd number of nitrogen atoms in the unit cell and nitrogen affinity to form molecular $\hbox {N}_2$; the dangling nitrogen left in the presence of the $\hbox {AsF}_6^-$ anion thus becomes problematic in terms of stability and computational convergence. Using PBE GGA and LDA functionals, the evolutionary search often comes to end without achieving the task as the majority of the structures cannot satisfy the system constraints. Only in one instance was LDA able to finish the search but without achieving polymerization of nitrogen into $\hbox {N}_5^+$ fragments (see Supplementary Fig. 3 for the best three structures obtained using the local approximation). While LDA lacks the delocalization required, PBE GGA overestimates the lattice parameters. PBEsol on the other hand is notorious for systematically lowering the lattice constants compared to other GGA functionals which improves equilibrium properties of densely packed solids and their surfaces^28^. With the volume shrinkage, polymerization of nitrogen becomes easier which explains PBEsol capability to obtain the desired $\hbox {N}_5^+$ polymorph. The obtained structure was further relaxed in a variable cell geometry optimization with PBEsol, PBE and LDA. The results are summarized in Table 1.

**Table 1:** Lattice parameters of the $\hbox {N}_5\hbox {AsF}_6$ structure in the space group H1 P1 at different levels of theory.

Functional	A (Å)	B (Å)	C (Å)	$\alpha ^{\circ }$	$\beta ^{\circ }$	$\gamma ^{\circ }$
PBEsol	5.4684	5.7817	6.9351	79.404	113.882	90.179
PBE	5.7038	5.9826	7.1294	77.512	114.158	90.338
LDA	5.2597	5.4179	6.4280	80.204	115.870	92.720

Similar search strategy was used for the $\hbox {N}_5\hbox {SbF}_6$ and $\hbox {N}_5\hbox {Sb}_2\hbox {F}_{11}$. Unfortunately the evolutionary search for $\hbox {N}_5\hbox {Sb}_2\hbox {F}_{11}$ was computationally too expensive and only a few structures satisfied the system constraints to progress towards better structures thus the halt of the algorithm. However, USPEX algorithm produced a similar triclinic structure for $\hbox {N}_5\hbox {SbF}_6$ with $\hbox {N}_5^+$ $\hbox {C}_{2V}$ and AsF6$^-$ $\hbox {O}_h$ symmetries preserved and with the following lattice constants: a = 6.2980 Å, b = 5.754 Å, c = 5.639 Å, $\alpha$ = 85.73$^{\circ }$, $\beta$ = 98.5760$^{\circ }$ and $\gamma$= 89.233$^{\circ }$. The crystal arrangement of $\hbox {N}_5\hbox {SbF}_{6}$ can be found in Supplementary Fig. 4.

### Stability, vibrational spectroscopy and phonon calculations

There are 12 atoms in the unit cell for $\hbox {N}_5\hbox {AsF}_6$, the phonon spectrum thus contains 36 branches (3 acoustic and 33 optical). These 36 branches correspond to 36 normal vibrational modes at the center of the first Brillouin zone $\Gamma$. Computational spectroscopy to get vibrational modes using the linear response methodology is only performed at the $\Gamma$ point. A tentative comparison with the observed IR signals regarding the $\hbox {N}_5^+$ of reference^9^ are summarized in Table 2 and the total vibrational data is provided in Supplementary Note 3. No negative frequencies are found upon imposing the acoustic sum rule. Also, excellent agreement can be found with the experimental Raman and infrared frequencies reported in the work of Christe et al.^9^. For instance, the strong experimental signals corresponding to the stretches of the $\hbox {N}_5^+$ cation at 2270 (mode 36) and 2210 (mode 35) cm$^{-1}$ are found in this work to be at 2332.90 and 2280.59 cm$^{-1}$ respectively. This good agreement further validates PBEsol capability in producing exact bond lengths for the $\hbox {N}_5^+$ terminal. Mode 34 on the other hand is largely overestimated. This is mostly due to the strong anharmonicity within DFT especially for the $\nu _8$ asymetric central stretch.

**Table 2:** Computational IR activity using the linear response approach at the PBEsol level of theory performed at $\Gamma$.

Mode	Assignment	Computed frequency	IR intensity	Observed frequencies^9^
33	$\hbox {N}_5^+$ $\nu _2$ symmetric central stretch	907.67	0.1443	872
34	$\hbox {N}_5^+$ $\nu _8$ asymmetric central stretch	1304.99	7.1052	1088
35	$\hbox {N}_5^+$ $\nu _7$ out-of-phase terminal stretches	2227.18	8.4356	2210
36	$\hbox {N}_5^+$ $\nu _1$ in-phase terminal stretches	2286.38	1.7218	2270

Four $\hbox {N}_{5}^{+}$-related normal modes observed in the work of Christe et al.^9^ are presented here for comparison. Frequency wavenumbers are in cm$^{-1}$ and IR intensities in (D/A)$^2$/amu.

**Figure 2:**
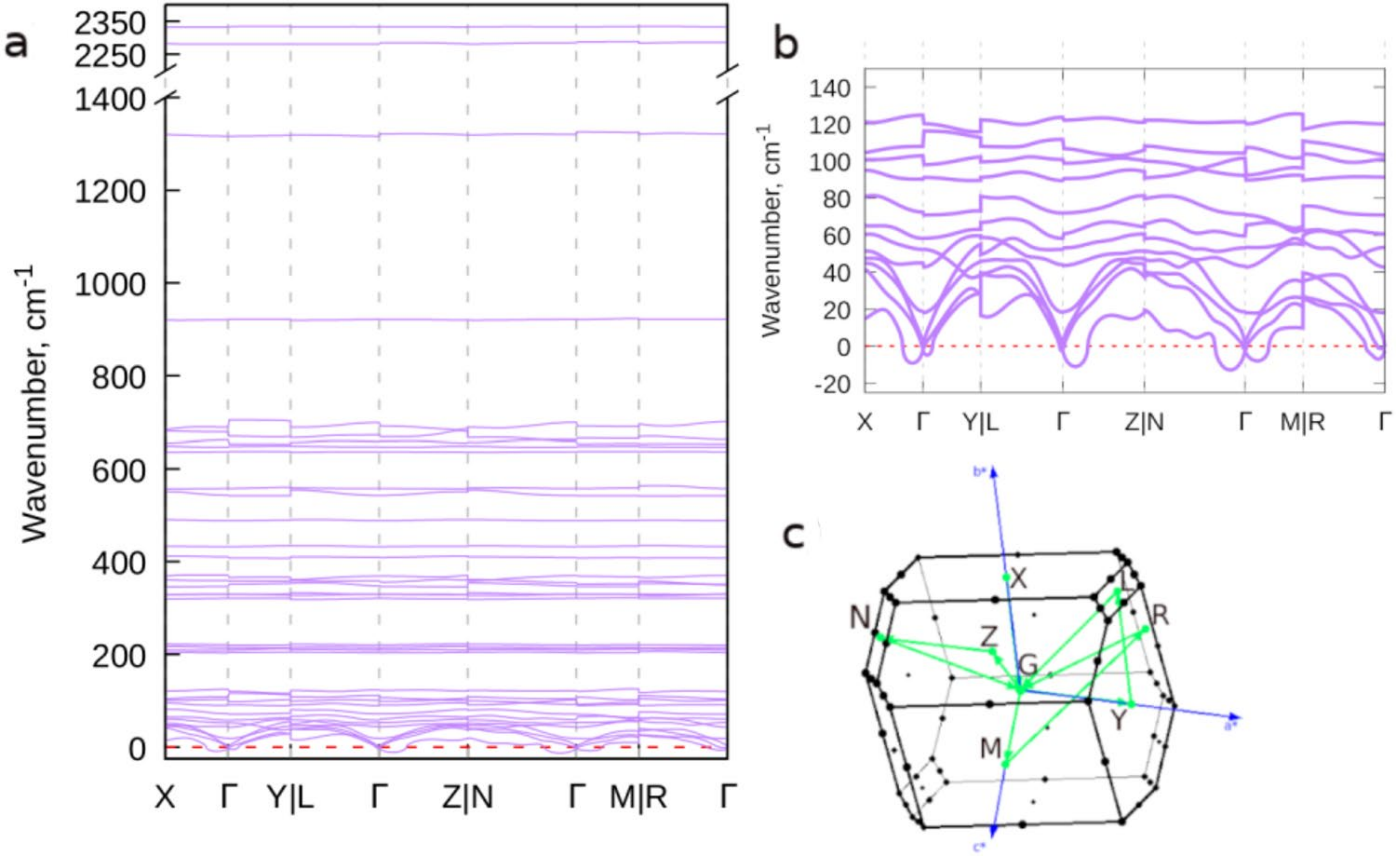
Calculated phonon dispersion of the $\hbox {N}_5\hbox {AsF}_6$ at 0 K and 0 GPa along the high symmetry points of the first Brillouin zone: (**a**) the full calculated phonon dispersion. (**b**) phonon dispersion for frequencies lower than 240 cm$^{-1}$ to allow for a clearer visualization of the soft phonon. (**c**) The high symmetry points path chosen in the first Brillouin zone. Unstable modes are shown in negative wavenumbers below the horizontal red line.

**Figure 3:**
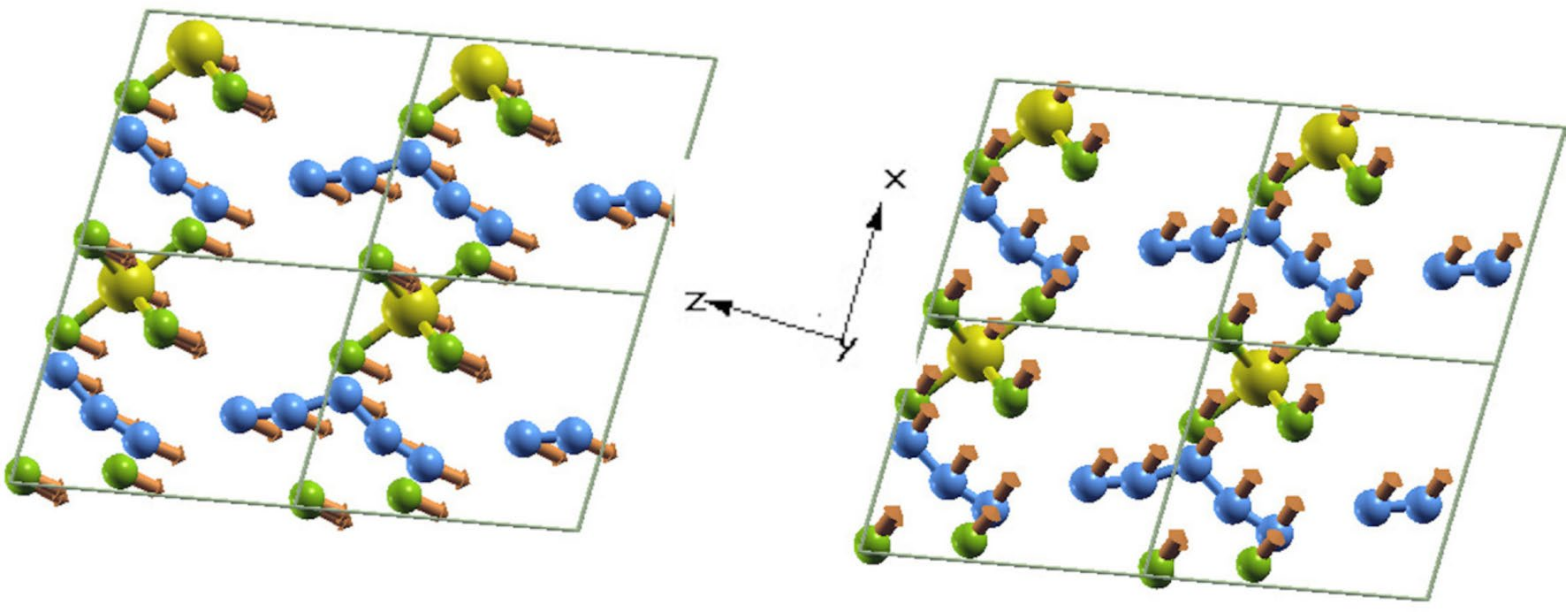
Crystal structure of the $\hbox {N}_5\hbox {AsF}_6$ at 0 K and 0 Gpa projected along [010] direction. The first (left) and second (right) accoustic phonon branches causing instability are librational and are depicted by forces on each atom. All forces are pointing in the same parallel thus perturbing the whole crystal in the same direction.

Figure 2 shows the phonon dispersion of N5$\hbox {AsF}_6$ based on the GGA approximation in the PBEsol formalism. We adopted the notations and crystallographic directions suggested in reference^30^ for such a triclinic structure of type TRI1b with the k-path: X-$\Gamma$-Y$|$ L-$\Gamma$-Z$|$ N-$\Gamma$-M$|$ R-$\Gamma$. Two partially unstable phonon modes as indicative from the negative frequencies developed can be observed along the high symmetry points of first the Brillouin zone. This softening is more severe towards $\Gamma$ point with the largest modulus in the Z-$\Gamma$ and N-$\Gamma$ directions. It is worth noting that this phonon branch corresponds to a collective motion of the crystal units in the same direction. In light of this finding, a trivial solution would be a structural phase transition to a lower symmetry structure to obtain more stable phonons. However, the determined H1 P1 space group for the $\hbox {N}_5\hbox {AsF}_6$ in this predictive search is already a low symmetry triclinic structure. Consequently, the marginal stability exhibited by $\hbox {N}_5\hbox {AsF}_6$ can be explained by the existence of these somewhat soft phonon branches. The computed phonon of this branch that causes instability of the structure at 0 K corresponds to the librational motion of the overall crystal in the same direction that is about 45 degrees from the [010] direction as shown in Fig. 3. This displacement is responsible for the perturbed stability of this compound up to an unknown temperature that should be within the experimental conditions of the synthesis and characterization of $\hbox {N}_5\hbox {AsF}_6$. It is important to note that ab initio calculations are conducted at zero temperature and zero pressure conditions while the synthesis and the spectroscopic data of reference^9^ were at ambient pressure and at temperatures of − 130 $^{\circ }$C for Raman and − 196.8 $^{\circ }$C for IR.

Moreover, high energy density materials such as $\hbox {N}_5\hbox {AsF}_6$ are highly explosive. Impact sensitivity and performance of such compounds are considered the key requirements towards their development. Upon impact, the first excitation takes place on the acoustic phonon at low wavenumbers, this causes energy to be transferred to higher energy phonon modes. For instance, bond breaking follows a stretching mode (about 1000 cm$^{-1}$ and higher) of the compound units before detonation can occur^31,32^. Softening of low frequency phonons has been observed in other high energy density materials, such as thalium azide $\hbox {TlN}_3$ below 240 K^33^. Therefore, $\hbox {N}_5\hbox {AsF}_6$ can be seen as a high explosive with higher sensitivity upon impact due to its thermally sensitive, soft acoustic phonons.

### Electronic structure and NBO investigation

**Figure 4:**
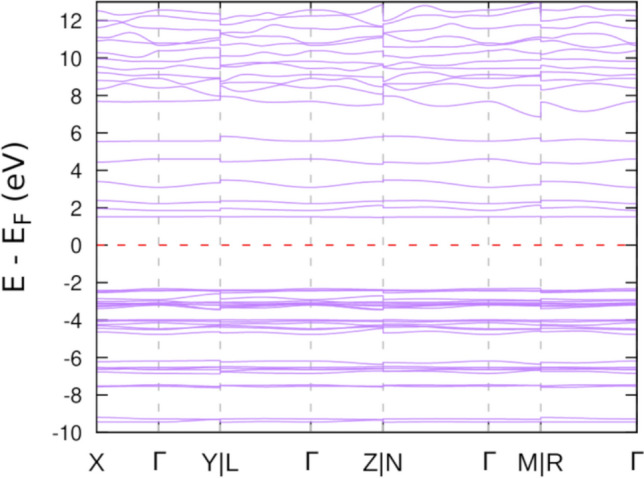
Electronic band structure of the $\hbox {N}_5\hbox {ASF}_6$ at 0 K and 0 GPa. The solid is an insulator with a direct band gap of 3.8 eV.

**Figure 5:**
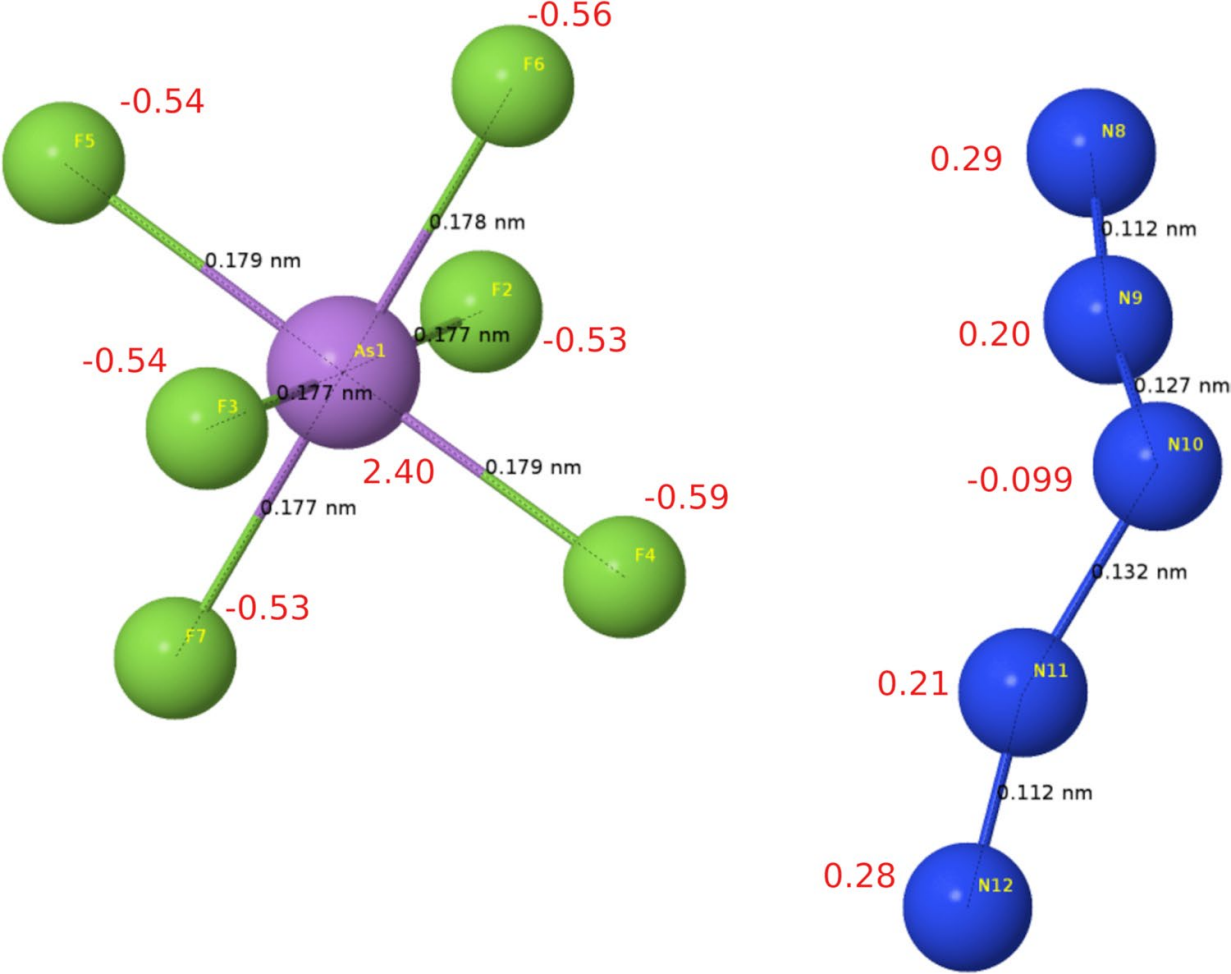
Bond lengths and natural charge of the two molecular units of $\hbox {N}_5\hbox {AsF}_6$ from natural bonding orbital (NBO) analysis. Further comparisons to the only known $\hbox {N}_5^+$ salt is shown in Table 3.

In Fig. 4, the electronic structure of $\hbox {N}_5\hbox {AsF}_6$ is plotted along the high symmetry points of the first Brillouin zone. Similar to most energetic materials, the $\hbox {N}_5\hbox {ASF}_6$ salt is an insulator. It has a large direct bandgap of 3.82 ev at the PBEsol level of theory. More importantly, Natural bonding orbital (NBO) analysis is more efficient in studying the intramolecular and intermolecular bonding that takes place at the molecular level. Hence its implementation in this investigation. $\hbox {N}_5^+$ stability was long believed to be attributed to its structural and electronic resonance^9,12^. $\hbox {N}_5^+$ and $\hbox {AsF}_6^-$ as optimized in the crystal were fed to the GamessUS^34^ density functional theory package (2019 R1 version) in combination with the natural bonding orbital method as implemented in nbo.6^35^ at the PBEsol level of theory. A summary of natural population analysis is shown in both Table 3 and Fig. 5e. This analysis provides the correct resonant $\hbox {N}_5^+$ configuration (A total of six resonant structures are debated to explain $\hbox {N}_5^+$ existence^9,12^). Partial natural charges on $\hbox {N}_5^+$ as shown in Fig. 5 and Table 3 in our work are within 7–11% difference in comparison to the work of Fau and Bartlet^36^. This discrepancy is mainly attributed to the electronic effects imposed by the neighbouring AsF6$^-$ as delivered from the solid in our investigation. NBO analysis also shows that $\hbox {N}_5^+$ polymorphism is largely driven by electronic considerations. From bond order analysis, the cation is encapsulated into stability through the strong triply bonded nitrogens from both ends (N8–N9 and N11–N12 with about 2.3 bond order in Fig. 5) despite the fragile central single bonds (N9–N10 and N10–N11 with about 1.1 bond order in Fig. 5). For comparison, the well-known azide salts such as $\hbox {NaN}_3$ which represent a class of high energy density materials have the azide anion $\hbox {N}_3^-$ stabilized through a similar encapsulation of the central atom electronically by resonant double and triple bonds within its linear chain.

**Table 3:** Comparative illustration of bond lengths (in Å) and bond angles of the $\hbox {N}_5^+$ between the $\hbox {N}_5\hbox {AsF}_6$ found in this investigation and that of the only $\hbox {N}_5^+$ salt known from the work of Vij et al^12^. The natural charge as obtained from Natural Population Analysis (NBO) is compared to the charge for the $\hbox {N}_5^+$ in the work of Bartlett et al.^36^ in the ideal gas phase $\hbox {C}_{2v}$ symmetry at the the NBO(B3LYP/aug-cc-pVDZ) level of theory^36^.

Bond	Bond length [this work]	Bond length [from Ref.^12^]	Bond order (BO) [this work]
N8–N9	1.12	1.102	2.312
N9–N10	1.27	1.295	1.117
N10–N11	1.32	1.303	1.077
N11–N12	1.12	1.107	2.341

	Bond angle [this work] (deg)	Bond angle [from Ref.^12^] (deg)	
N9–N10–N11	114.4	111.2	
N8–N9–N10	166.1	168.1	
N10–N11–N12	161.8	168.1	

Atom number	Charge [PBEsol ]	Charge [B3LYP/6-31++G$$^{**}$$]	Charge [ from Ref.^36^]
As1	2.40	2.57	
F2	− 0.54	− 0.58	
F3	− 0.54	− 0.58	
F4	− 0.59	− 0.63	
F5	− 0.53	− 0.57	
F6	− 0.55	− 0.59	
F7	− 0.53	− 0.56	
N8	0.29	0.31	0.33
N9	0.20	0.21	0.22
N10	− 0.098	− 0.12	− 0.11
N11	0.20	0.22	0.22
N12	0.28	0.31	0.33

Compared to the only known crystal structure among all $\hbox {N}_5^+$ salts which is $\hbox {N}_5\hbox {Sb}_2\hbox {F}_{11}$ from reference^12^, excellent agreement was found for the intramolecular bond lengths and angles. A summary of this comprehensive comparison can be found in Table 3 with a focus on the $\hbox {N}_5^+$ fragment only since the counter anion in $\hbox {N}_5\hbox {Sb}_2\hbox {F}_{11}$ is not the same and involves two $\hbox {SbF}_6^-$ units sharing a bridging fluorine. Similar to end-on addition of $\hbox {N}_5^+$ and $\hbox {N}_5^-$ salts to obtain compounds with higher nitrogen content, earlier attempts to investigate addition of $\hbox {N}_5^+$ and $\hbox {N}_3^-$ salts was conducted by Fau and Bartlett^36^ in the gas phase. Isolating a covalently bonded $\hbox {N}_8$ is found to be difficult because decomposition is energetically more favourable. However, we believe that taking the whole crystal of $\hbox {N}_5^+$ into consideration is necessary since a large lattice energy within a solid-state structure was long believed to allow such addition^36^.

## Conclusion

In this systematic evolutionary search for the high energy density material $\hbox {N}_5\hbox {ASF}_6$, we were able to unravel its elusive crystal structure and the cause of its marginal stability. Our investigation confirms the polymeric nitrogen configuration in this crystal to be of $\hbox {C}_{2V}$ symmetry. The center of the Brillouin zone exhibits no negative vibrational modes and is in good agreement with the spectroscopic data. A partial softening of two of the low frequency acoustic phonon modes takes place at the vicinity of the center from different directions inside the Brillouin zone. The collective displacement of the two molecular units of this crystal in the same direction are responsible for energy transfer to higher frequency phonon, thus the explosive instability of the crystal. NBO theory also unraveled the true resonant $\hbox {N}_5^+$ structure that explains its stability through an electronic encapsulation by strong triple bonding on both ends of this polymeric nitrogen. Over three decades of polymeric nitrogen investigations, many compounds were hypothesized to exist in low and high pressure domains. However, the traditional routes to find these compounds relied on intuition and trial and error to assess the possibility of their existence. In this work, evolutionary genetic predictions prove to be more efficient and require little human interaction. Moreover, PBEsol functionals within DFT is proven to be computationally more suitable than other functionals for providing a more accurate description of polymeric nitrogen structures. This work is thus believed to be a forerunner for more implementation of PBEsol in dealing with polymeric nitrogen compounds in the future. The approach implemented is also assessed to successfully predict other high energy density materials, such as extended carbon-oxygen systems.

## Methods

USPEX (version 9.4.4), a powerful evolutionary algorithm for stable structure predictions^23–25^ starting from only their stocheometry was used. For $\hbox {N}_5\hbox {AsF}_6$ under investigation, an initial pool size of 30 random crystal structures was created for the first generation. In subsequent generations, 50% of the structures were produced by heredity, 20% randomly and the remaining 30% were equally produced by permutation, softmutation and lattice mutation. Geometry optimization for each structure was conducted in four steps, the first two steps allowing only the ions to move without changing the unit cell. In the last 2 steps, ions and the containing cell were both allowed to vary during the relaxation. More details of the simulation run are given in Supplementary Note 4. Quantum ESPRESSO^37,38^, was used as the ab initio density functional theory (DFT^26,27^) engine to carry all the structural optimizations, the electronic structure and the phonon calculations. Different DFT functionals were tried for the evolutionary search including LDA^27^ and GGA^29^. Only the Perdew–Burke–Ernzerhof (PBE) functional in the framework of PBEsol^28^ GGA satisfied the search criteria. Other functionals often led to the halt of the evolutional methodology of USPEX because the majority of crystal structures did not handle the system constraints. The strictest resolution in the fourth relaxation step during USPEX search had a resolution of 2$\pi \times$ 0.10 Å$^{-1}$ and a kinetic energy cutoff of 680 eV. A higher cutoff of 1088 eV was imposed after the best structure was obtained in the remaining calculations. Further geometry optimization was carried out using LDA, PBE and PBEsol to assess the impact of the approximation used on the compound lattice constants. The vibrational frequencies by computing the IR activity was carried out at the PBE level of theory but with a norm conserving pseudopotential at $\Gamma$. The choice is due to the limitation in Quantum espresso’s implementation of of the DFT perturbation theory in evaluating the normal modes along with their Raman and IR intensities. However, the full phonon calculations were conducted using density functional perturbation theory^39^ with PBEsol. The electronic Brillouin zone integration as well as phonon calculations were performed on a Monkhorst-Pack 12$\times 12\times$12 k-point meshes and a 2$\times 2\times$2 mesh of phonon vectors for the dynamical matrix computation. The coordinates of the two molecular units making the crystal were fed to GamessUS^34^ linked with nbo.6^35^ to carry out the natural bonding orbital (NBO) theory analysis without boundary conditions and without any further geometry optimization. Partial charges were then extracted from this analysis. The true bonding within $\hbox {N}_5^+$ was also extracted from the bond order obtained with this NBO analysis. The NBO investigation was performed at the PBE level of theory with PBEsol and B3LYP/6-31++G. Xcrysden^40^, VESTA^41^ and Jmol^42^ were used for the visualization of the results and the plots of this manuscript.

## Supplementary information

Supplementary material 1 (docx 239 KB)
