# Supplementary material for: Predicting the crystal structure of N5AsF6 high energy density material using ab initio evolutionary algorithms
Source: Sci Rep. 2021 Apr 12;11:7874. doi: 10.1038/s41598-021-86855-2 (PMC8041836; doi:10.1038/s41598-021-86855-2)
Supplement: Supplementary file 1 — Supplementary material 1 (docx 239 KB) [file 41598_2021_86855_MOESM1_ESM.docx]

**Predicting the crystal structure of N_5_AsF_6_ high energy density material using ab initio evolutionary algorithms**

**El Mostafa Benchafia^1^, Xianqin Wang^2^, Zafar Iqbal^3^ and Sufian Abedrabbo^1,4^**
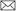


^1^University, Department of Physics, Abu Dhabi, UAE.
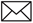
Email: sufian.abedrabbo@ku.ac.ae

^2^New Jersey Institute of Technology, Department of Chemical,Biological and Pharmaceutical Engineering, Newark, NJ 07102, USA

^3^New Jersey Institute of Technology, Department of Chemistry and Environmental Science, Newark, NJ 07102, USA

^4^University of Jordan, Department of Physics, Amman, Jordan

**Supplementary Note-1**

Journalist Malcolm W. Browne wrote a piece for the New York Times with the title “New Nitrogen Ion Carries Warning: Handle with Care New York Times. Malcolm W. Browne February 2, 1999, Section F, Page 2” The link to this newspaper article can be found at:

<https://www.nytimes.com/1999/02/02/science/new-nitrogen-ion-carries-warning-handle-with-care.html>

**The crystal arrangement of N_5_Sb_2_F_11_**

The only N_5_ salt with XRD data available is that of N_5_Sb_2_F_11_. In the work of Vij et al.^1^, the synthesis of the N_5_SbF_6_ was achieved and was used as a precursor for N_5_Sb_2_F_11_. While the N_5_^+^ C_2v_ arrangment is preserved in both salts, N_5_Sb_2_F_11_ has a shared fluorine atom between two SbF_6_ anions.


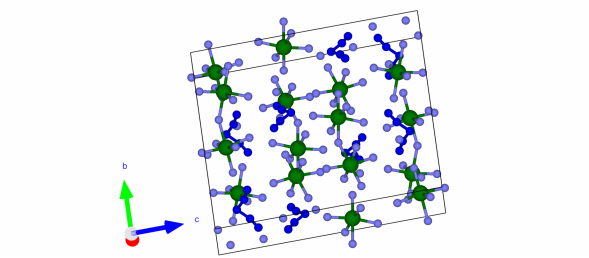


**Supplementary Figure S1.** The crystal arrangement in N_5_Sb_2_F_11_ as obtained from its data and structure Refinement. Notice the bridging Fluorine atom shared between two SbF_6_ units. The structure is C2/c monoclinc with unit cell dimensions a=10.913(8) Å, b =12.654(8) Å, c=16.675(11) Å, α=90◦ , β=104.715(18)◦ and γ= 90◦.

**The CRYSTAL ARRANGEMENT IN N_5_AsF_6_**

**
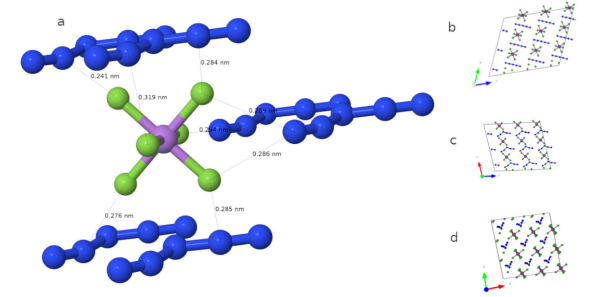
**

**Supplementary Figure S2.** The crystal arrangement in N_5_AsF_6_ as obtained from the USPEX predictive search using PBEsol. a- The AsF6^-^ anion between two N_5_^+^ planes, the third N_5_^+^ planes pass through the center of AsF6^-^ with the closest distance between nitrogen and fluorine at 2.41 Å. b-d projections of 3×3 N_5_AsF_6_ supercell along the a, b and c axes respectively.

**Supplementary Note-2:** **N_5_AsF_6_ Crystallographic Information File (CIF)**

Crystallographic Information File (CIF) of the best structure as obtained with the USPEX code. Structural parameters after geometry reoptimization at different levels of theory using stricter cutoffs are slightly different than the raw CIF data.

data_findsym-STRUC-

_symmetry_space_group_name_H-M 'P 1'

_symmetry_Int_Tables_number 1

_cell_length_a 5.43800

_cell_length_b 5.76600

_cell_length_c 6.92600

_cell_angle_alpha 66.83800

_cell_angle_beta 100.36300

_cell_angle_gamma 90.14800

loop_

_atom_site_label

_atom_site_type_symbol

_atom_site_fract_x

_atom_site_fract_y

_atom_site_fract_z

_atom_site_occupancy

N N 0.4800 0.4349 -0.0619 1.0000

N N 0.3186 -0.4890 -0.2361 1.0000

N N 0.2178 -0.4133 -0.4075 1.0000

N N 0.3621 0.2759 0.2792 1.0000

N N 0.3916 0.3543 0.1093 1.0000

As As -0.1409 -0.0018 -0.2033 1.0000

F F 0.0379 0.1297 -0.0341 1.0000

F F 0.0204 -0.3038 -0.0408 1.0000

F F 0.0920 0.0676 -0.3618 1.0000

F F -0.3165 -0.1332 -0.3716 1.0000

F F -0.2986 0.2998 -0.3646 1.0000

F F -0.3688 -0.0738 -0.0399 1.0000

**USPEX with the local density approximation (LDA)**


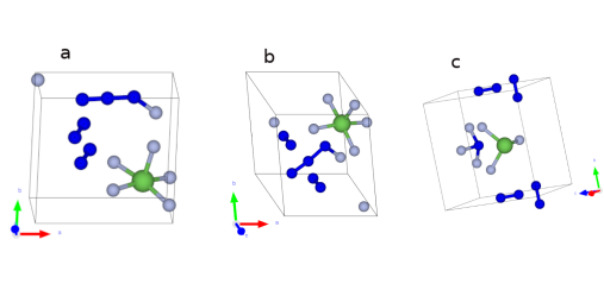


**Supplementary Figure S3.** The crystal arrangement in N_5_AsF_6_ as obtained from the USPEX predictive search with the local density approximation of DFT. a-c The three best energetically favorable structures attained. LDA fails to obtain N_5_^+^ polymerization. Instead, molecular N_2_ and at best FN_3_ units were obtained. Interestingly, experimental attempts to isolate FN_5_ from thermolysis of N_10_SnF_6_ in the work of Wilson et al.^2^ were unsuccessful as only FN_3_ and N_2_ decomposition products were produced.

**The crystal arrangement in N_5_SbF_6_**

**
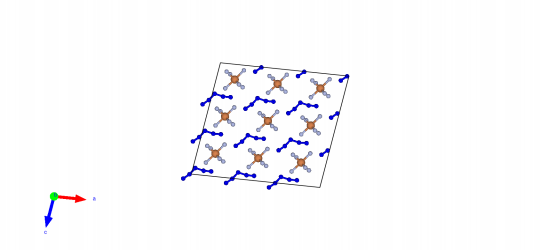
**

**Supplementary Figure S4.** The crystal arrangement in a 3×3 N_5_SbF_6_ units as obtained from the USPEX predictive search using PBEsol. The structure is triclinc with unit cell dimensions a = 6.2980 Å, b = 5.754 Å, c = 5.639 Å, α = 85.730 ^◦^ , β = 98.576 ^◦^ and γ = 89.233 ^◦^.

**Supplementary Note-3: Vibrational frequencies**

Table S1. Computational IR activity using the linear response approach at the PBEsol level of theory performed at Γ. No negative frequencies were found. Imposing the acoustic sum rule brought all acoustic modes to zero frequencies. Frequency wavenumbers are in cm^−1^ and IR intensities in (D/A)^2^/amu. Four N_5_^+^-related normal modes observed in the work of Christe et. al^3^ are presented here for comparison.

| Mode and Assignment | Computed frequency | R intensity | Observed frequencies^3^ |
| --- | --- | --- | --- |
| 1  2  3  4  5  6  7  8  9  10  11  12  13  14  15  16  17  18  19  20  21  22  23  24  25  26  27  28  29  30  31  32  33 **ν_2_ symmetric central stretch**  34 **ν_8_ asymmetric central stretch**  35 **ν_7_ out-of-phase terminal stretches**  36 **ν_1_ in-phase terminal stretches** | 0.00  0.00  0.00  62.90  86.41  105.38  130.91  149.08  155.54  191.07  206.65  213.81  221.54  257.07  268.52  277.32  349.69  355.54  363.36  365.78  369.24  384.11  402.33  430.83  484.54  535.73  554.98  623.07  633.20  641.30  648.89  654.77  907.67  1304.99  2227.18  2286.90 | 0.00  0.00  0.00  0.1399  0.3878  0.1095  0.1184  0.0725  0.4465  0.3058  0.1541  0.0863  0.1018  0.2446  0.0469  0.2428  2.7392  0.9066  2.8047  1.0541  0.4734  3.3653  0.1994  0.0208  0.0090  0.0210  0.0325  2.4472  6.7966  6.1715  0.1330  5.6891  0.1443  7.1052  8.4356  1.7218 | 872  1088  2210  2270 |

**Supplementary Note-4: USPEX parameters**

The first generation in the USPEX run consists of 30 structures created randomly. The same number of structures was produced in each generation afterwards. Heredity consists of 50% of the structures produced starting from the second generation and every other generation that follows. 20% of the structures are again produced randomly, 10% by permutation, 10% by softmutation and 10% by lattice mutation. The Maximum number of generations allowed for the simulation was set to 100 but the USPEX algorithm converged in this investigation at generation 27 at maximum from different runs. The simulation was set to stop if the best structure did not change after 20 generations. The fraction of the generation that should be used to produce the generation that follows was kept at the default value of 0.7. Care was taken to perform ab initio relaxations with DFT in 4 steps (the variable cell approach was conducted in the last two steps while only atoms could vary in the first two steps within the cell).

**References**

1. Vij, A. et al. Polynitrogen chemistry. synthesis, characterization, and crystal structure of surprisingly stable fluoroantimonate salts of N_5_^+^ . J. Am. Chem. Soc. 123, DOI: 10.1021/ja010141g (2001).

2. Dr, W. et al. Polynitrogen chemistry: Preparation and characterization of (N5)2SnF6, N5SnF5, and N5B(CF3)4. Chem. - A. Eur. J. 9, 2840 – 2844, DOI: 10.1002/chem.200304973 (2003).

3. Christe, K. O., Wilson, W. W., Sheehy, J. A. & Boatz, J. A. N_5_^+^: A novel Homoleptic Holynitrogen Ion as a High Energy Density Material. Angewandte Chemie Int.
